# Optogenetics, physiology, and emotions

**DOI:** 10.3389/fnbeh.2013.00169

**Published:** 2013-11-19

**Authors:** Alexxai V. Kravitz, Antonello Bonci

**Affiliations:** ^1^Diabetes, Endocrinology, and Obesity Branch, National Institute of Diabetes, Digestive, and Kidney DiseasesBethesda, MD, USA; ^2^National Institute of Drug AbuseBaltimore, MD, USA

**Keywords:** optogenetics, emotions, anxiety, depression, reward, physiological, synchrony

Optogenetics is a powerful tool for investigating causal links between neural circuits and behavior. In recent years, optogenetic studies have expanded into the emotional realm, elucidating new facts about the circuits that underlie anxiety, depression, and reward. One caveat with investigating this realm is that emotional responses can be non-linear. Reward is pleasurable to a point, beyond which it can produce mania, an anxious, and unpleasant state. Consistent with this, stimulant drugs that increase dopaminergic function are reinforcing across a limited dose range, above which they are no longer reinforcing, presumably because of anxiogenic effects (Ettenberg and Geist, 1991, 1993; Yang et al., 1992; Deroche et al., 1997). Many published optogenetic studies have not examined potential non-linearities in the relationship between neural activity and the behavior being studied, nor identified where on such a curve their optogenetic manipulation is acting. In addition, the state a neural system achieves during optogenetic stimulation may not necessarily reside on a physiological curve at all, as optogenetics can drive firing parameters outside of physiological ranges (Figure 1). We believe that further consideration of this point may lead to more accurate insights into the relationships between neural activity, emotions, and behavior.

**Figure 1:**
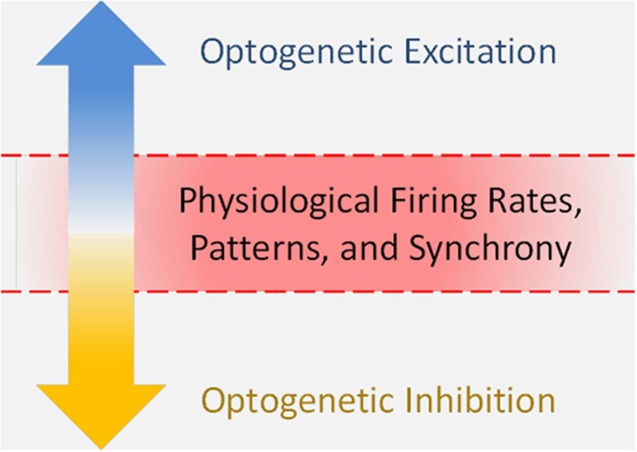
**Optogenetics can drive firing parameters outside of physiological ranges**.

Largely for technical reasons, early optogenetic studies did not record neural activity during behavioral manipulations, but instead used slice physiology to validate their manipulations. These studies linked increased activity in specific circuitry to wakefulness (Adamantidis et al., 2007), movement (Aravanis et al., 2007; Gradinaru et al., 2007, 2009; Kravitz et al., 2010), reinforcement (Tsai et al., 2009; Lobo et al., 2010; Stuber et al., 2011; Britt et al., 2012; Kravitz et al., 2012; Liu et al., 2012), anxiety (Tye et al., 2011), and feeding (Aponte et al., 2011), among others. In these studies, slice experiments demonstrated that light could evoke spiking specifically in the target cell types, but could not address how spiking was altered during the behavioral manipulation. The technical barriers to recording from awake animals during optogenetic stimulation have been bridged in recent years, as many researchers have integrated optogenetics with awake *in vivo* recording (Cardin et al., 2010; Royer et al., 2010; Anikeeva et al., 2012; Cohen et al., 2012; Kravitz et al., 2012; Warden et al., 2012; Tye et al., 2013). This allows for comparisons between spiking during spontaneous behavioral and emotional states and optogenetically evoked states. However, defining and quantifying the relevant parameters for such comparisons is still not trivial. Important parameters include firing rates, spatial and temporal firing patterns of individual neurons, and synchrony among populations of neurons. Investigating these parameters in both the spontaneous and stimulated conditions will improve our ability to interpret optogenetic studies, especially when the relationship between firing rates and behavior is not linear.

The efficacy of optogenetically-evoked firing depends on many factors, including viral expression and optical transmission efficiency. In spite of the variability of these factors between laboratories, we believe that many studies are driving firing rates at the high end of, or above, rates achieved under spontaneous conditions. For example, when striatal neurons were tested with a range of stimulation intensities (0.1–3.0 mW), 1 mW was found to achieve nearly maximal firing rates of light-activated neurons within ~0.5 mm of the stimulation fiber (Kravitz et al., 2012). Other studies have stimulated striatal neurons with higher intensities of light (up to 20 mW) (Lobo et al., 2010; Britt et al., 2012; Tai et al., 2012), likely saturating the firing of many neurons. Excitable structures such as hippocampus and cortex are susceptible to seizure activity, which defines a hard upper limit to usable stimulation intensities. Seizures have been induced with ~20 mW stimulation of the hippocampus at 10–20 Hz (Osawa et al., 2013), and alluded to with ~10–20 mW stimulation of the motor cortex at 20 Hz(Gradinaru et al., 2009). These stimulation paradigms are not much stronger than what has been used to stimulate hippocampal cells to re-activate fear memory (~9 mW at 20 Hz) (Liu et al., 2012), stimulate cortical cells to facilitate movement (~10–20 mW, constant or pulsed) (Aravanis et al., 2007; Gradinaru et al., 2009), and stimulate amygdala pyramidal cells to facilitate fear conditioning (~30 mW at 20–50 Hz) (Johansen et al., 2010). Again, without *in vivo* recordings it is difficult to relate different stimulation paradigms to actual firing rates. Still, it is still fair to conclude that most published studies have focused on stimulating near the high end of usable stimulation intensities, which could result in firing outside of that which occurs spontaneously. Moving forward, it may also be worth exploring the low end of stimulation intensity, to examine the effects of more subtle changes in firing which may relate more closely to physiological conditions, or at least reveal a more complete relationship between firing and behavior.

In addition to firing rates, neural systems contain information in spatial (the physical location of the neurons in the brain) and temporal codes (changes in firing rate of these neurons over time) (Pouget et al., 2000; Jortner et al., 2007; Ainsworth et al., 2012). With regard to these spatial and temporal codes, one potentially surprising result of published optogenetic stimulation studies is that most stimulation paradigms have produced relatively normal behavioral responses while presumably saturating and overpowering these codes. This may suggest that rate coding is more important than temporal or spatial coding, although this conclusion is difficult to reconcile with the ubiquity and wealth of information contained in these codes (Pouget et al., 2000; Jortner et al., 2007; Ainsworth et al., 2012). Alternatively, it is possible that stimulation of certain cell groups is “permissive,” rather than “informative,” in which case the exact pattern or even intensity of firing may not be relevant. As a final possibility, spatial, and temporal codes could be preserved during optogenetic stimulation, riding “along the top” of the stimulated increase in firing rate. While it is not possible to replicate spatial codes with single light sources, new advances with multiple micron-scale light sources (Grossman et al., 2010; Kim et al., 2013a,b), or holographic methods for delivering light (Lutz et al., 2008) may allow for more physiological manipulations of both spatial and temporal coding. This said, such multi-site light sources would need to achieve cellular resolution to truly replicate physiological spatial codes.

Finally, optogenetic stimulation can alter correlations and synchrony among groups of neurons. Most published optogenetic studies have used pulsed stimulation to drive neural activity, which effectively synchronizes populations of neurons at the optical stimulation frequency (Aravanis et al., 2007; Gradinaru et al., 2009; Johansen et al., 2010; Stuber et al., 2011; Tye et al., 2011; Anikeeva et al., 2012; Liu et al., 2012; Burguiere et al., 2013; Osawa et al., 2013). There is growing consensus that aberrant synchronization is a pathological hallmark of many neurological diseases including Parkinson's disease, epilepsy, autism, and schizophrenia (Uhlhaas and Singer, 2006; Brown, 2007). Parkinson's disease is characterized by aberrant synchronization of multiple basal ganglia structures including the subthalamic nucleus (STN) in the beta band (~10–30 Hz) (Brown, 2007). Optically stimulating fibers innervating the STN at 20 Hz (at ~10 mW) worsened parkinsonian motor deficits, while stimulating these same fibers at 130 Hz (also at ~10 mW) alleviated these deficits (Gradinaru et al., 2009). In motor cortex, 130 Hz stimulation again alleviated parkinsonian deficits, while 20 Hz had no effect. In this way, synchronizing a structure at a particular frequency can have effects beyond altering firing rates. One way to mitigate potential issues with synchrony may be to stimulate circuits with a range of frequencies, including irregular patterns of stimulation that avoid synchronizing a structure at a specific frequency (Tai et al., 2012). Alternatively, more subtle stimulation such as low power constant illumination or step-function opsins may avoid synchronizing the cells at any particular frequency, while still increasing the output of target cells (Kravitz et al., 2010).

Optogenetic inhibition can side step the above problems of over-stimulation, although it can still induce aberrant synchronization from both optogenetic inhibition and potential rebound spiking. However, as the principal neurons in many brain structures are low firing and optogenetic inhibition is often incomplete *in vivo* (Aravanis et al., 2007; Gradinaru et al., 2009; Royer et al., 2012; Calu et al., 2013; Kim et al., 2013a,b), it may be more difficult to push neurons below their physiological firing rates with optogenetic inhibition. This may be an inherent benefit of optogenetic inhibition for studies that aim to manipulate activity of specific cells to rescue or mimic physiological states. However, other loss-of-function experiments can benefit from inhibiting neurons, irrespective of physiological ranges. Complementary methodologies such as designer receptors exclusively activated by designer drugs (DREADDs) can also investigate the contribution of specific cell types while avoiding aberrant patterns of synchronization (Ferguson et al., 2011; Krashes et al., 2011). Ideally both inhibition and stimulation can be examined in the same system [e.g., (Stuber et al., 2011; Tye et al., 2011, 2013; Britt et al., 2012; Chaudhury et al., 2013; Chen et al., 2013)], in combination with *in vivo* electrophysiology to test whether firing parameters during the manipulations are physiologically relevant. This impressive trifecta has been accomplished by a few studies to date [e.g., (Jennings et al., 2013; Tye et al., 2013)].

The above issues are central for optogenetic experiments that make inferences about neural activity during naturally occurring emotional or behavioral states. However, a second type of experiment sets aside concerns regarding physiological patterns of activity, and aims to stimulate or inhibit with the sole objective of interfering with, and thus mitigating unwanted behavior for therapeutic utility. Here, understanding the relationship between physiological firing of a structure and the emotion or behavior of interest is not as relevant as reducing the unwanted behavior, quite like how deep brain stimulation (DBS) or trans-cranial magnetic stimulation (TMS) can mitigate pathological conditions in neurological disorders without necessarily replicating normal physiological conditions (Miocinovic et al., 2013). In this vein, optogenetic manipulations have been therapeutic for reducing symptoms associated with movement disorders (Gradinaru et al., 2009; Kravitz et al., 2010), depression (Chaudhury et al., 2013; Tye et al., 2013), and compulsive cocaine seeking (Chen et al., 2013).

Regardless of the specific stimulation parameters or methodology, optogenetics has revolutionized neuroscience and offers unprecedented mechanistic and therapeutic opportunities to understand and treat neurological and psychiatric diseases. Quantification of the relationships between physical brain states and perceptual states, as well as firing during optogenetic manipulations will further this progress by facilitating easier quantitative comparisons between studies, reduce the potential for spurious conclusions, and ultimately provide more meaningful insights into the relationships between neural circuits, emotions, and behavior.

## Acknowledgment

We acknowledge Drs. Marc Reitman and Mike Krashes for helpful comments.
